# Central Serous Chorioretinopathy Diagnosed by Emergency Practitioner‐Performed Ocular Point‐Of‐Care Ultrasonography

**DOI:** 10.1002/ajum.70005

**Published:** 2025-04-14

**Authors:** Christian P. Pappas, Matthew Watson, Christopher Harrington, Katherine Masselos

**Affiliations:** ^1^ Faculty of Medicine and Health The University of New South Wales Randwick New South Wales Australia; ^2^ Department of Ophthalmology Prince of Wales Hospital Randwick New South Wales Australia; ^3^ Department of Emergency Medicine Prince of Wales Hospital Randwick New South Wales Australia

**Keywords:** central serous chorioretinopathy, diagnostic techniques, emergency medicine, ophthalmological, ultrasonography

## Abstract

**Introduction:**

Central serous chorioretinopathy (CSCR) is a common cause of acute, monocular vision loss amongst men aged 40–50 years. Diagnosis is typically multimodal, requiring advanced ophthalmic imaging. These techniques are not readily available in acute care settings.

**Methods:**

We report the first case of CSCR diagnosed by an emergency practitioner–performed ocular point‐of‐care ultrasonography (POCUS).

**Results:**

CSCR was identified by the presence of a dome‐shaped, hypoechoic elevation of the neurosensory retina in association with a hypoechoic band posterior to the retinal pigment epithelium. The diagnosis was confirmed following ophthalmic referral. The patient was managed conservatively with routine observation and risk factor modification.

**Conclusion:**

We describe the first reported use of emergency practitioner‐performed ocular POCUS to identify findings suggestive of CSCR, a common cause of acute monocular vision loss among working‐aged men. Although this case demonstrates the evolving utility of ocular ultrasound in emergent eye presentations, further research is needed to define the technique's role in the early evaluation of CSCR.

**Summary**

- Central serous chorioretinopathy (CSCR) is a common cause of acute vision loss amongst men of working age.
- Diagnosis is typically multimodal, requiring a combination of dilated posterior segment examination and advanced ophthalmic imaging, which are not readily available in emergency departments to which patients often initially present.
- We review the first reported case of CSCR diagnosed in the emergency department by ocular point‐of‐care ultrasonography (POCUS), emphasising typical sonographic findings and the evolving use of ocular ultrasound in emergent eye presentations.

## 1 Case Report

**FIGURE 1:**
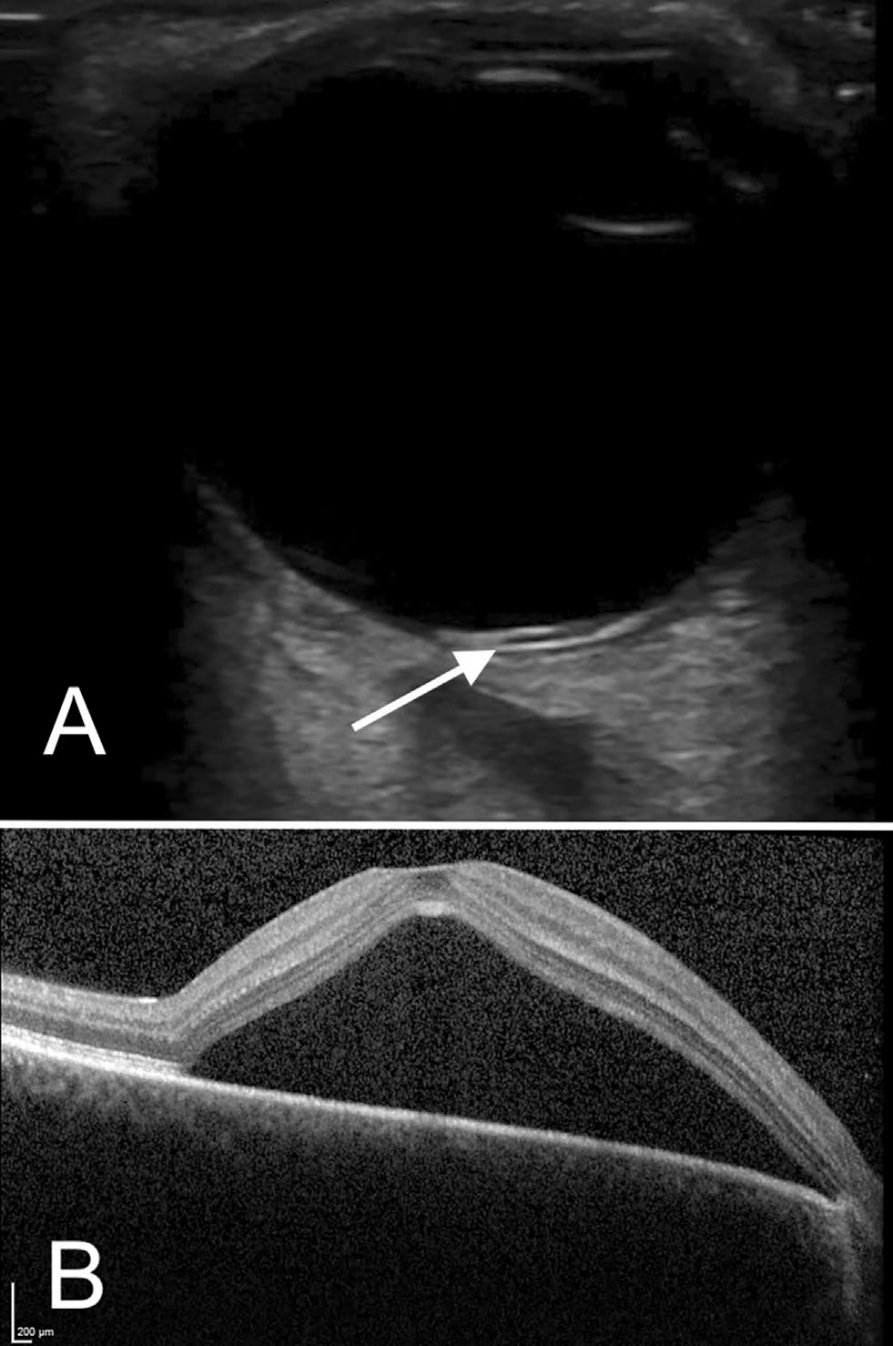
(A) High frequency transverse ultrasonography of the right eye with a linear probe. The RPE is denoted by the white arrow. Serous retinal detachment is seen as an immobile, dome‐shaped non‐echogenic region anterior to the RPE, with a corresponding band of non‐echogenicity posteriorly, potentially representing choroidal hyper‐permeability. (B) Optical coherence tomography of the right eye demonstrates a large sub‐foveal serous retinal detachment extending to involve the superior macula.

A 39‐year‐old male presented to the emergency department with a 2‐week history of atraumatic monocular right eye visual changes. He reported an initial episode of flashes followed by the development of a central scotoma, with intact peripheral vision. He had a background of anxiety, alcohol use disorder, and cluster A personality traits. Neuro‐ophthalmological examination was performed including assessment of Snellen visual acuity, visual fields by confrontation, cranial nerve examination (with careful examination for a relative afferent pupillary defect), anterior segment slit lamp examination (including fluorescein staining), intraocular pressures and direct fundoscopy. Pertinent findings included decreased right eye visual acuity 6/60, with intact visual acuity 6/6 in the left eye. Right eye visual fields by confrontation demonstrated impaired central with intact peripheral vision. An emergency practitioner‐performed ocular POCUS was performed using a GE Healthcare Technologies Inc. (Chicago, USA) Venue with a L12n‐RS linear probe with frequencies 3.5–12 MHz. The ‘nerve’ pre‐set was used with depth 2–3 cm to permit visualisation of the retina and retro‐bulbar optic nerve. Gain and time gain compensation (TGC) were initially optimised to reduce internal vitreous echoes, emphasising the vitreoretinal interface. Gain was subsequently increased to exclude hyperechoic intravitreal material associated with vitreous haemorrhage or rhegmatogenous retinal detachment. Static and dynamic cineloop views in the transverse, longitudinal and sagittal planes were obtained. Ocular POCUS demonstrated non‐echogenic bands anterior and posterior to the retinal pigment epithelium (RPE) within the right eye, suggestive of central serous chorioretinopathy (CSCR) (Figure 1).

The patient was subsequently referred for further ophthalmic assessment with a provisional diagnosis of CSCR based on patient history and risk factors, examination signs and suggestive ocular POCUS findings. Differential diagnoses at the time of referral included optic neuritis, posterior vitreous detachment, papilloedema, or maculopathy including Valsalva retinopathy. Optical coherence tomography (OCT) in the ophthalmology clinic confirmed the presence of serous macular detachment with associated subretinal fluid and gravitational changes on fundus autofluorescence (FAF), consistent with right eye CSCR. Similar, less pronounced changes were identifiable in the left eye. The patient was managed conservatively with routine observation and risk factor modification.

## 2 Ethics Statement

The patient provided informed consent to the publication of images and data in this study. This study was reviewed by the South Eastern Sydney Local Health District Human Research Ethics Committee, Low Negligible Risk sub‐committee, who confirmed that formal ethical review was not required in accordance with the NHMRC National Statement (updated 2018).

## 3 Discussion

Sight‐threatening presentations to emergency departments are common, accounting for 9% of all ocular presentations [1]. If not identified and managed promptly, these patients are at risk of developing a lasting visual deficit. Within this context, emergency practitioner‐performed ocular POCUS has found increasing use as an accessible, cost‐effective, and rapid diagnostic tool, with an established role in the diagnosis of rhegmatogenous retinal detachment, vitreous detachment, vitreous haemorrhage, intraocular foreign body, lens dislocation and globe rupture. Ocular POCUS also finds use as a screening tool for non‐specific posterior segment pathology. In contrast, the finding of central serous chorioretinopathy (CSCR), a common cause of acute monocular vision loss, by emergency practitioner‐performed ocular POCUS has not been previously reported.

Central serous chorioretinopathy is a detachment of the neurosensory retina from the RPE in the setting of subretinal fluid accumulation, thought attributable to RPE dysfunction, hyperpermeability, and thickening of the underlying choroid. CSCR is a frequent cause of acute monocular vision loss amongst working‐aged men [2], with an estimated age‐adjusted incidence of 9.9 and 1.7 cases per 100,000 person‐years for males and females respectively [3], with peak onset between 40 and 50 years [2]. Affected individuals classically present with monocular loss of central visual acuity, with other complaints including monocular micropsia (perceiving objects as smaller, or more distant than they really are), metamorphopsia (wavy distortion of linear objects) and reduced colour sensitivity. The most significant risk factor for the development of CSCR is exogenous glucocorticoid use, including local and systemic formulations, with an odds ratio of 37.1 [4]. Although other significant risk factors include endogenous hypercortisolism (Cushing's syndrome), uncontrolled systemic hypertension, *Helicobacter pylori* infection, alcohol use, psychiatric and lifestyle factors are of particular relevance [2]. Indeed, type A personality traits are recognised risk factors for the development of CSCR, while a history of psychiatric illness has been associated with a greater risk of recurrences [2]. Stressful life events, shift work and circadian rhythm disruptions are also recognised risk factors [2].

Although the diagnosis of CSCR is typically multimodal, using a combination of OCT, FAF, indocyanine green, and intravenous fluorescein angiography, these techniques are not readily available in acute care settings and are typically done in an ophthalmology clinic. As our case demonstrates, ocular POCUS may have a novel role in the diagnosis of CSCR in emergency department settings. Optimal image quality for ocular tissues of the posterior segment requires a high‐frequency (7.5–14 MHz) linear probe. The image capture protocol may include cineloop views in the transverse, longitudinal and sagittal planes. Gain and TGC should be optimised such that the posterior chamber is hypoechoic, and post‐acoustic enhancement impairing the view of the optic nerve sheath is minimised. Serous macular detachment is identified by the presence of a dome‐shaped non‐echogenic elevation of the neurosensory retina [5]. Although similar subtle findings can be seen in the setting of Valsalva retinopathy [6] and cystoid macular oedema (CMO), a prospective observational case series of 5 patients with unilateral CSCR, 5 patients with unilateral CMO, and 10 age‐matched control subjects found the additional presence of a non‐echogenic linear band posterior to the RPE in all eyes affected by CSCR [5]. This is thought to represent choroidal hyperpermeability, suggesting a diagnosis of CSCR [5]. Although the early use of ocular ultrasound to identify the condition may assist in risk stratification and triaging the urgency of ophthalmic review, further research is needed to quantify test characteristics and the risk of over‐diagnosis in emergency medicine settings.

Management of CSCR broadly aims to preserve the outer neurosensory retinal layers and achieve resolution of underlying subretinal fluid accumulation [2]. Acute cases are often managed conservatively with risk factor modification and observation, where spontaneous resolution can occur within 3–6 months [2]. In contrast, refractory or recurrent cases are often treated with photodynamic therapy and focal laser photocoagulation among other methods [2]. Although the visual prognosis in CSCR is relatively favourable, between 30% and 52% of untreated individuals experience recurrent disease within 1 year [2].

## 4 Conclusion

In summary, we review the first reported use of emergency practitioner‐performed ocular POCUS to identify findings suggestive of CSCR, a common cause of acute, monocular vision loss amongst working‐aged men. Although diagnosis is typically multimodal requiring advanced ophthalmic imaging techniques, further research may define a role for emergency practitioner‐performed POCUS in the early evaluation of this condition.

## Author Contributions

The authorship listing conforms with the journal's authorship policy, and all authors are in agreement with the content of the submitted manuscript.

## Ethics Statement

This study was reviewed by the South Eastern Sydney Local Health District (SESLHD) Human Research Ethics Committee, Low Negligible Risk sub‐committee, who confirmed that formal ethical review was not required in accordance with the NHMRC National Statement (updated 2018).

## Consent

The patient has provided informed consent to the publication of images and data.

## Conflicts of Interest

The authors declare no conflicts of interest.
